# Association of telomere shortening with low back pain and the potential mediating role of smoking: A cross-sectional study

**DOI:** 10.1097/MD.0000000000045806

**Published:** 2025-11-07

**Authors:** Yao Liu, Min Huang, Jiaxin Li, Bin Pu

**Affiliations:** a Department of Orthopedics, Suining City Traditional Chinese Medicine Hospital, Suining, Sichuan, China.

**Keywords:** chronic pain, low back pain, mediation analysis, smoking, telomere length

## Abstract

Chronic pain, particularly low back pain (LBP), is common among older adults and may be influenced by cellular aging. Telomere length (TL) has been suggested as a marker for age-related susceptibility to musculoskeletal pain, but evidence in elderly populations is limited. This study examined the association between TL and chronic pain, including LBP, and explored the potential mediating role of smoking. We conducted a cross-sectional analysis of 2070 participants aged ≥ 60 years from National Health and Nutrition Examination Survey 1999 to 2002. TL was measured using quantitative polymerase chain reaction, and pain outcomes were self-reported via questionnaires. Weighted multivariable logistic regression models assessed associations between TL and chronic pain. Mediation analysis was performed to evaluate the potential role of smoking in the TL–LBP relationship. Shorter TL was significantly associated with higher odds of LBP (odds ratios = 0.77, 95% confidence interval: 0.63–0.95), while no significant associations were observed for neck or joint pain. TL was inversely associated with smoking, which in turn was related to higher odds of LBP. Mediation analysis suggested that smoking may partially mediate the association between TL and LBP, explaining approximately 60.9% of the total effect. Shorter TL is associated with LBP in older adults, with smoking potentially acting as a partial mediator. These findings highlight TL as a potential biomarker for lumbar spine-related pain and support smoking cessation as a possible intervention target. Longitudinal studies are needed to further clarify causal pathways and site-specific mechanisms.

## 1. Introduction

Chronic pain represents a pervasive health burden among the aging population, exerting profound effects on physical functioning, quality of life, and healthcare utilization.^[1,2]^ Low back pain (LBP), joint pain, and neck pain are among the most common complaints in older adults, with prevalence estimates exceeding 76.2% in community-dwelling elderly individuals.^[3]^ These conditions not only compromise mobility and independence but also contribute to multimorbidity, psychological distress, and increased healthcare costs.^[4]^ Despite extensive epidemiological investigations, the biological determinants of chronic pain in late life remain incompletely understood.

Telomere length (TL), a molecular marker of cellular aging, has gained increasing attention as a potential biomarker for age-related diseases and functional decline.^[5–7]^ Telomeres, the repetitive DNA–protein structures capping chromosome ends, progressively shorten with each cell division and are susceptible to oxidative stress and inflammation.^[8,9]^ Accelerated telomere attrition has been implicated in cardiovascular disease,^[10]^ diabetes,^[11]^ neurodegeneration,^[12]^ and mortality,^[13]^ yet its role in chronic pain remains underexplored. Notably, pain is strongly associated with systemic inflammation and stress-related pathways,^[14,15]^ suggesting that TL shortening may reflect biological processes underlying pain susceptibility. However, existing evidence on the relationship between TL and chronic pain has been limited and inconsistent, with most studies focusing on joint disorders^[16]^ or generalized pain,^[17]^ and few specifically addressing LBP in elderly populations.

Smoking, a well-established behavioral factor influencing both telomere dynamics and pain outcomes, may serve as a mechanistic link in this context. Smoking accelerates telomere erosion through heightened oxidative stress and inflammatory activation,^[18,19]^ while also being recognized as a major risk factor for musculoskeletal pain^[20,21]^ and intervertebral disc degeneration.^[22,23]^ Nevertheless, no population-based study to date has systematically investigated whether smoking mediates the association between TL and chronic pain, particularly LBP, in older adults.

Against this backdrop, we leveraged data from the National Health and Nutrition Examination Survey (NHANES) 1999 to 2002, a nationally representative sample of the U.S. population, to examine the associations between TL and chronic pain outcomes in adults aged 60 years and older. Specifically, we aimed to evaluate whether TL is associated with neck pain, LBP, and joint pain, and to explore the potential mediating role of smoking in the relationship between TL and LBP. By integrating molecular biomarkers with epidemiological data, this study provides novel insights into the biological underpinnings of pain in the aging population and highlights potential targets for prevention and intervention strategies.

## 2. Methods

### 2.1. Study design and population

**Figure 1.**
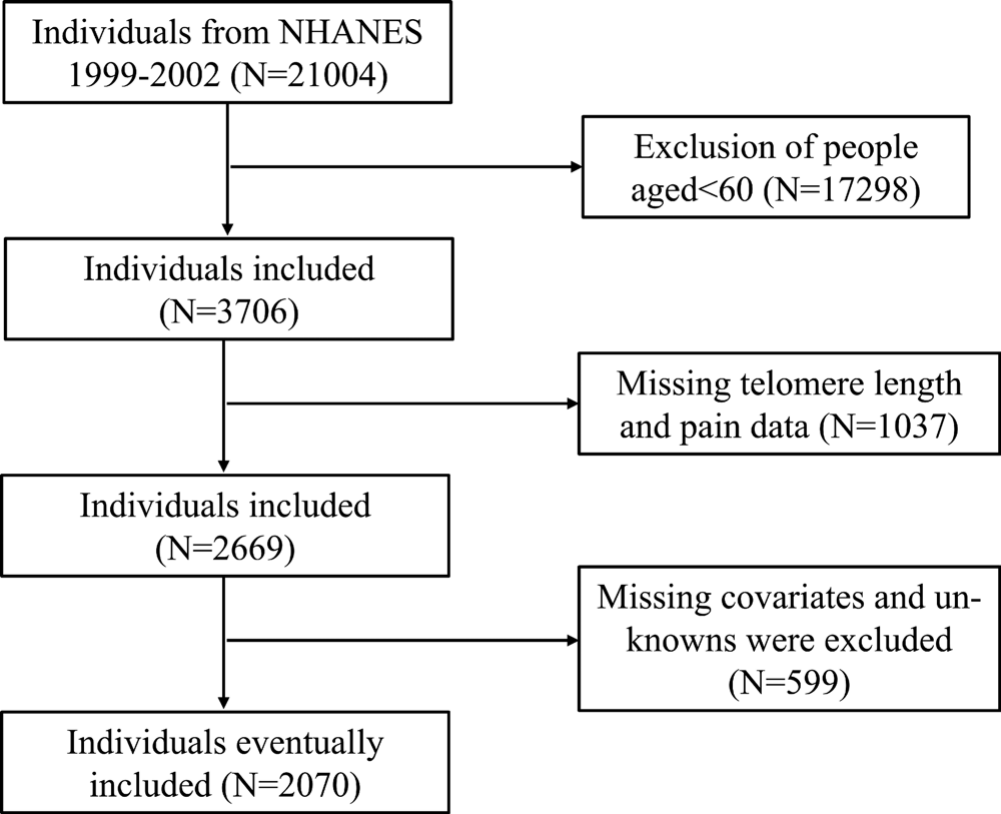
Flowchart to demonstrate the selection of participants in this study.

We conducted a cross-sectional analysis using data from the NHANES 1999 to 2002, a nationally representative survey of the non-institutionalized U.S. population that employs a multistage, stratified sampling design. Standardized interviews, examinations, and biospecimen collection were performed in mobile examination centers.^[24]^ NHANES protocols were approved by the National Center for Health Statistics (NCHS) Research Ethics Review Board, and all participants provided written informed consent.^[25]^ For this analysis, we included adults aged 60 years and older, excluding those with missing data on TL, chronic pain, or key covariates. Finally, among 21,004 participants, through strict eligibility criteria, a total of 2070 participants were included in the study (Fig. 1).This study followed the Strengthening the Reporting of Observational Studies in Epidemiology (STROBE) guidelines for cross-sectional studies.

### 2.2. Assessment of TL

TL was measured in leukocyte DNA obtained from NHANES blood samples. The assay was conducted at the University of California, San Francisco, using a quantitative polymerase chain reaction method and expressed as the ratio of telomere repeat copy number to single-copy gene number (T/S ratio). Each sample was assayed in replicates with reference DNA controls to adjust for inter-assay variability. Runs or outliers not meeting quality standards were excluded, and the mean value was used for analysis. The inter-assay coefficient of variation was 6.5%.^[26,27]^

### 2.3. Assessment of chronic pain

Chronic pain outcomes were derived from the NHANES Miscellaneous Pain Questionnaire. Participants were asked whether they had experienced neck pain or LBP during the past 3 months, and joint pain during the past 12 months. Individuals who responded “yes” were classified as having the corresponding pain. It is important to note that recall periods differed: 3 months for LBP/neck pain and 12 months for joint pain.”

### 2.4. Covariate assessment

Following established methodologies in prior studies, several covariates were included in the analyses. Demographic variables comprised age (continuous), sex (male/female), and race, categorized as Mexican American, non-Hispanic White, non-Hispanic Black, or other race. Socioeconomic indicators included education level (less than high school, high school, and college or above) and the poverty-to-income ratio (PIR), categorized as poor (<1), near poor (1–3), or not poor (≥3) in accordance with NHANES definitions.^[28]^ Marital status, used as a proxy for social support, was dichotomized as living alone versus living with someone. Lifestyle factors included smoking status (never, former, and current smokers) and alcohol consumption, defined based on the NHANES questionnaire as consuming at least 12 alcoholic drinks in the past year (yes/no). Physical health status was represented by body mass index (BMI, continuous, kg/m²). In addition, 2 comorbidities were considered: diabetes and hypertension, both coded as binary variables (yes/no).

### 2.5. Statistical analyses

All statistical analyses were conducted using R software (version 4.3.3, R Foundation for Statistical Computing, Vienna, Austria). Given the complex multistage, stratified, and clustered sampling design of NHANES and the availability of Mobile Examination Center sampling weights, weighted survey design objects (svydesign) incorporating Mobile Examination Center weights, stratification (SDMVSTRA), and clustering (SDMVPSU) were used to ensure representativeness of the U.S. elderly population. Continuous variables were assessed for normality and presented as means ± standard deviations if normally distributed, or as medians and interquartile ranges if non-normally distributed, whereas categorical variables were presented as counts and percentages. Differences between sexes were evaluated using the Wilcoxon rank-sum test for continuous variables and the Chi-square test for categorical variables.

Associations between TL and chronic pain outcomes, including neck pain, LBP, and joint pain, were examined using weighted multivariable logistic regression models. Model 1 was unadjusted; Model 2 adjusted for age, sex, and race; Model 3 further adjusted for education level, PIR, alcohol consumption, BMI, hypertension, and diabetes. All models employed survey weights to reflect the true characteristics of the U.S. elderly population.

To explore the mediating role of smoking in the relationship between TL and LBP, mediation models were constructed to estimate the effect of TL on smoking, the effect of smoking on LBP, and the direct effect of TL on LBP. β coefficients, odds ratios (ORs), and 95% confidence intervals (CIs) were calculated, with adjustment for the aforementioned covariates. All statistical tests were 2-sided, and statistical significance was defined as *P* < .05.

## 3. Results

### 3.1. Participant characteristics

The study population comprised 2070 U.S. community-dwelling adults aged ≥ 60 years (1076 male, 994 female), with mean ages of 70.90 ± 7.56 and 71.16 ± 7.88 years, respectively: a difference that is modest yet statistically significant (*P* < .001). Strikingly, disparities were observed between sexes in socioeconomic and health-related characteristics: PIR, marital status, educational level, smoking and alcohol consumption, TL, and hypertension prevalence all differed significantly (all *P* < .05), indicating pronounced sexual dimorphism in these domains. Notably, BMI and diabetes prevalence did not differ significantly between men and women (*P* > .05) (Table 1).

**Table 1:** Characteristics of participants aged 60 years and older from the NHANES 1999–2002.

Characteristic	Overall	Male	Female	*P*
	(N = 2070)	(N = 1076)	(N = 994)	
Age, mean (SD)	71.02 (7.51)	70.90 (7.56)	71.16 (7.88)	<.001
Race, No. (%)				.009
Mexican American	417 (2.9)	215 (2.9)	202 (2.8)	
Non-Hispanic White	1223 (83.0)	652 (84.8)	571 (81.6)	
Non-Hispanic Black	303 (6.5)	151 (5.9)	152 (7.0)	
Other	127 (7.6)	58 (6.4)	69 (8.6)	
Marital status, No. (%)				<.001
Live with someone	1321 (65.7)	850 (82.5)	471 (51.4)	
Live alone	749 (34.3)	226 (17.5)	523 (48.6)	
Education level, No. (%)				.001
Below high school	853 (28.9)	454 (29.0)	399 (28.8)	
High School	495 (30.1)	226 (25.2)	269 (34.3)	
College or above	722 (41.0)	396 (45.8)	326 (36.9)	
PIR, No. (%)				<.001
Poor	311 (11.6)	147 (7.9)	164 (14.7))	
Near poor	1010 (45.8)	504 (42.0)	506 (49.0)	
Not poor	749 (42.7)	425 (31.1)	324 (36.3)	
Smoking status, No. (%)				<.001
Never smoker	959 (46.7)	345 (31.1)	614 (60.1)	
Former smoker	866 (41.2)	579 (55.4)	287 (29.1)	
Current smoker	243 (12.1)	150 (13.5)	93 (10.9)	
Alcohol consumption, No. (%)				<.001
Yes (had at least 12 alcohol drinks/1 year)	1284 (61.0)	839 (77.6)	445 (46.9)	
No	786 (39.0)	237 (22.4)	549 (53.1)	
BMI (kg/m^2^), mean (SD)	28.28 (5.36)	27.94 (4.68)	28.66 (5.99)	.123
Telomere length (kb), No. (%)	5.43 (0.52)	5.37 (0.48)	5.49 (0.55)	.001
Diabetes, No. (%)				.279
Yes	357 (14.3)	196 (15.4)	161 (13.4)	
No	1713 (85.7)	880 (84.6)	833 (86.6)	
Hypertension, No. (%)				.001
Yes	1067 (50.9)	514 (46.1)	553 (54.9)	
No	1003 (49.1)	562 (53.9)	441 (45.1)	
Low back pain, No. (%)				.006
Yes	766 (39.1)	367 (35.2)	399 (42.4)	
No	1304 (60.9)	709 (64.8)	595 (57.6)	
Joint pain, No. (%)				.002
Yes	1085 (53.9)	516 (51.4)	569 (58.4)	
No	985 (46.1)	560 (48.6)	425 (41.6)	
Neck pain, No. (%)				.326
Yes	383 (18.5)	178 (17.1)	205 (19.7)	
No	1687 (81.5)	898 (82.9)	789 (80.3)	

Normally distributed continuous variables were presented as means with standard deviations (SDs), while categorical variables were expressed as proportions. The Wilcoxon rank-sum test was used for comparisons of continuous variables, and chi-square tests were applied for categorical variables. Sample weights from the Mobile Examination Center were adjusted to ensure the results’ representativeness for the US civilian population.

BMI = body mass index; kb = kilobase pairs; NHANES = National Health and Nutrition Examination Survey; OR = odds ratio; PIR = poverty-to-income ratio.

### 3.2. Association between TL and chronic pain

In our comprehensive analyses, neither neck pain nor joint pain was significantly associated with TL, suggesting that TL may not be directly implicated in these pain types. In contrast, a robust inverse association emerged between TL and LBP: each decrement in TL corresponded to a significantly elevated odds of LBP in the unadjusted model (Model 1: OR = 0.83, 95% CI: 0.69–0.99, *P* = .045), with the association persisting after adjustment for age, sex, and race (Model 2: OR = 0.78, 95% CI: 0.65–0.94, *P* = .016), and remaining statistically significant even in the fully adjusted model controlling for education level, PIR, alcohol consumption, BMI, hypertension, and diabetes (Model 3: OR = 0.77, 95% CI: 0.63–0.95, *P* = .032) (Table 2).

**Table 2:** Associations between telomere length and chronic pain.

Characteristics	Model 1 OR (95% CI)	*P*-value	Model 2 OR (95% CI)	*P*-value	Model 3 OR (95% CI)	*P*-value
Neck pain	0.97 (0.71–1.31)	.829	0.83 (0.59–1.17)	.308	0.80 (0.58–1.11)	.218
LBP	0.83 (0.69–0.99)	.045*	0.78 (0.65–0.94)	.016*	0.77 (0.63–0.95)	.032*
Joint pain	1.01 (0.83–1.21)	.950	0.98 (0.8–1.19)	.839	0.95 (0.76–1.18)	.640

Model 1: no covariates were adjusted.

Model 2: age, sex, and race were adjusted.

Model 3: age, sex, race, education level, PIR, alcohol consumption, BMI, hypertension and diabetes were adjusted.

BMI = body mass index; LBP = low back pain; OR = odds ratio; PIR = ratio of family income to poverty; TL = telomere length.

* *P*-value < .05.

### 3.3. Mediation by smoking

**Figure 2.**
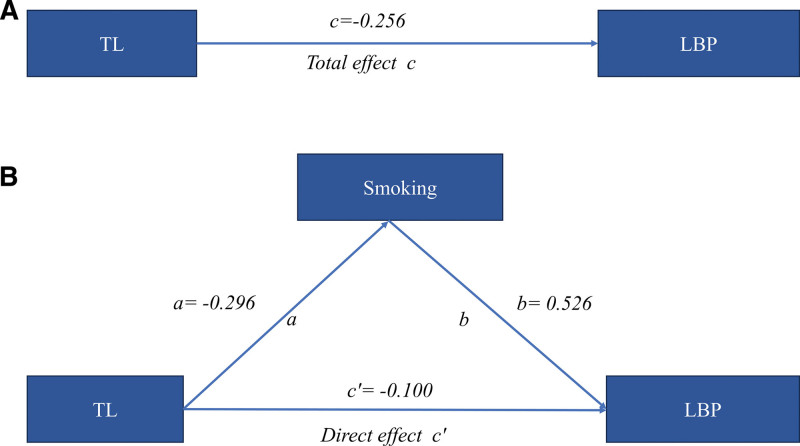
Total effect of TL on LBP (A) and mediation model for smoking (B). (A) The total effect of TL on LBP (*c*) was estimated using weighted multivariable logistic regression, adjusting for age, sex, race, education level, PIR, alcohol consumption, BMI, diabetes, and hypertension. (B) The total effect was decomposed into: (i) indirect effect via smoking, calculated using the product of coefficients method (*a* × *b*), where a represents the effect of TL on smoking and b represents the effect of smoking on LBP, both adjusted for the same set of covariates, and (ii) direct effect of TL on LBP (*c*′ = *c* − *a* × *b*). BMI = body mass index; LBP = low back pain; PIR = poverty-to-income ratio; TL = telomere length.

Moreover, mediation analysis delineated both direct and indirect pathways through which TL influences LBP. Shorter TL was associated with increased LBP risk (total effect β = –0.256, 95% CI: –0.486 to –0.026; OR = 0.774, 95% CI: 0.615–0.974; *P* = .032). TL was inversely related to smoking (β = –0.296, 95% CI: –0.586 to –0.007; OR = 0.744, 95% CI: 0.557–0.994; *P* = .046), and smoking in turn heightened LBP risk (β = 0.526, 95% CI: 0.187–0.865; OR = 1.692, 95% CI: 1.205–2.376; *P* = .007) (Table 3). The indirect effect through smoking (*a* × *b* = -0.156) accounted for 60.9% of the total effect, indicating that smoking serves as a partial mediator in the pathway linking TL shortening to LBP (Fig. 2).

**Table 3:** Smoking status as a mediator of the relationship between TL and LBP.

Exposure	Outcome	β (95% CI)	OR (95% CI)	*P*-value
TL	LBP	-0.256 (-0.486 to -0.026)	0.774 (0.615 to 0.974)	.032*
TL	smoking	-0.296 (-0.586 to -0.007)	0.744 (0.557 to 0.994)	.046*
Smoking	LBP	0.526 (0.187 to 0.865)	1.692 (1.205 to 2.376)	.007*

All models were adjusted for age, sex, race, education level, PIR, alcohol consumption, BMI, hypertension, and diabetes. The association between smoking status and LBP was additionally adjusted for TL.

β values with 95% confidence intervals (CI), odds ratios (OR) with 95% CI, and *P*-values are presented.

BMI = body mass index; LBP = low back pain; PIR = ratio of family income to poverty; TL = telomere length.

* *P*-value < .05.

## 4. Discussion

In this nationally representative cohort of U.S. adults aged 60 years and older, we found that shorter TL was significantly associated with a higher risk of LBP, whereas no significant associations were observed between TL and either neck pain or joint pain. Importantly, mediation analysis revealed that smoking partially mediated the relationship between TL and LBP, accounting for approximately 60.9% of the total effect. These findings indicate that shorter TL is associated with a higher likelihood of LBP, and that smoking-related biological processes may play a role in the pathway linking telomere shortening with pain susceptibility.

Our findings provide novel evidence linking TL to LBP in older adults, a population where epidemiological data have been scarce. Previous studies have mostly focused on osteoarthritis^[29,30]^ or generalized musculoskeletal pain,^[17]^ with limited exploration of lumbar spine–specific pain. TL shortening may reflect not only systemic biological aging but also local susceptibility of the lumbar spine to age-related degeneration. The lumbar region is uniquely burdened by continuous mechanical loading, making it prone to intervertebral disc degeneration.^[31,32]^ At the molecular level, shortened telomeres impair the proliferative capacity of disc cells and promote a senescence-associated secretory phenotype, which accelerates extracellular matrix breakdown and structural instability.^[33]^ In facet joints, telomere shortening in cartilage and subchondral bone similarly fosters degenerative changes and pain sensitization.^[34]^ These biological processes offer a plausible explanation for the robust association we observed between TL and LBP, underscoring TL as a molecular hallmark that bridges systemic aging with lumbar spine-specific musculoskeletal disorders in the elderly.

The null associations between TL and neck pain or joint pain warrant further discussion. Neck pain often arises from muscle injury, posture-related factors, or cervical spondylosis,^[35]^ which may be less strongly influenced by systemic biological aging markers compared with LBP. Similarly, joint pain in older adults is heterogeneous, with etiologies ranging from osteoarthritis to inflammatory arthritis and trauma,^[36,37]^ which may dilute the association with TL. Moreover, our pain assessment relied on self-reported questionnaires, which could lead to misclassification, particularly for joint pain with variable onset and duration. These explanations highlight the importance of considering pain site-specific pathophysiology when investigating molecular biomarkers of pain.

The mediating role of smoking provides additional mechanistic insight. Smoking has been firmly established as a contributor to disc degeneration,^[38]^ impaired bone metabolism,^[39]^ and chronic back pain.^[40]^ At the molecular level, smoking accelerates telomere erosion through oxidative stress and inflammation.^[19]^ Our results indicate that smoking partially mediates the relationship between TL and LBP, suggesting that behavioral and biological processes linked to smoking may account for a substantial proportion of the observed association.

From a translational perspective, these results underscore the value of TL as a potential biomarker for chronic pain risk stratification and highlight smoking cessation as a modifiable intervention target. Given the high prevalence and socioeconomic burden of LBP in aging populations, integrating telomere biology into pain prevention strategies may open new avenues for precision medicine and public health interventions.

One major strength of our study is that it is the first, to our knowledge, to examine the relationship between TL and LBP in older adults while incorporating mediation analysis to elucidate the behavioral role of smoking. Nevertheless, some caveats should be noted. This study has several limitations. First, its cross-sectional design precludes causal inference, and prospective studies are needed to determine whether telomere attrition precedes pain onset. Second, pain outcomes were self-reported, with different recall periods for LBP/neck pain (3 months) and joint pain (12 months), which may have introduced recall bias; moreover, the absence of standardized pain scales in NHANES limited the precision of pain severity assessment. Third, TL reflects systemic cellular aging but may not fully capture local degenerative processes in the lumbar spine that directly contribute to pain. Finally, residual confounding cannot be excluded, as important factors such as depression and physical activity were not measured in NHANES.

In conclusion, our study provides novel evidence that telomere shortening is significantly associated with LBP, but not with neck or joint pain, in older adults. Smoking emerged as a key mediator of this relationship, linking cellular aging with pain vulnerability. These findings suggest that shorter TL is associated with LBP in older adults, with smoking appearing to partially mediate this relationship. Future longitudinal and mechanistic studies are needed to further clarify the site-specific role of telomere attrition in musculoskeletal pain.

## Acknowledgments

We thank the National Center for Health Statistics (NCHS) for making NHANES data publicly available. We also appreciate the participants of NHANES for their valuable contributions.

## Author contributions

**Data curation:** Yao Liu, Jiaxin Li.

**Funding acquisition:** Bin Pu.

**Methodology:** Jiaxin Li.

**Project administration:** Bin Pu.

**Resources:** Yao Liu, Jiaxin Li.

**Software:** Min Huang.

**Visualization:** Min Huang.

**Writing – original draft:** Yao Liu, Min Huang, Bin Pu.

**Writing – review & editing:** Yao Liu, Jiaxin Li, Bin Pu.
